# Benchmark Dose Estimation for Cadmium-Induced Renal Tubular Damage among Environmental Cadmium-Exposed Women Aged 35–54 Years in Two Counties of China

**DOI:** 10.1371/journal.pone.0115794

**Published:** 2014-12-23

**Authors:** Jia Hu, Mei Li, Tian-xu Han, Jian-wei Chen, Lin-xiang Ye, Qi Wang, Yi-kai Zhou

**Affiliations:** 1 MOE Key Lab of Environment and Health, Institute of Environmental Medicine, School of Public Health, Tongji Medical College, Huazhong University of Science & Technology, Wuhan, China; 2 Department of Epidemiology and Biostatistics, School of Public Health, Tongji Medical College, Huazhong University of Science & Technology, Wuhan, China; University of Sao Paulo Medical School, Brazil

## Abstract

**Background:**

A number of factors, including gender, age, smoking habits, and occupational exposure, affect the levels of urinary cadmium. Few studies have considered these influences when calculating the benchmark dose (BMD) of cadmium. In the present study, we aimed to calculate BMDs and their 95% lower confidence bounds (BMDLs) for cadmium-induced renal tubular effects in an age-specific population in south-central China.

**Methods:**

In this study, urinary cadmium, β2-microglobulin, and N-acetyl-β-D-glucosaminidase levels were measured in morning urine samples from 490 randomly selected non-smoking women aged 35–54 years. Participants were selected using stratified cluster sampling in two counties (counties A and B) in China. Multiple regression and logistic regression analyses were used to investigate the dose-response relationship between urinary cadmium levels and tubular effects. BMDs/BMDLs corresponding to an additional risk (benchmark response) of 5% and 10% were calculated with assumed cut-off values of the 84^th^ and 90^th^ percentile of urinary β2-microglobulin and N-acetyl-β-D-glucosaminidase levels of the controls.

**Results:**

Urinary levels of β2-microglobulin and N-acetyl-β-D-glucosaminidase increased significantly with increasing levels of urinary cadmium. Age was not associated with urinary cadmium levels, possibly because of the narrow age range included in this study. Based on urinary β2-microglobulin and N-acetyl-β-D-glucosaminidase, BMDs and BMDLs of urinary cadmium ranged from 2.08 to 3.80 (1.41–2.18) µg/g cr for subjects in county A and from 0.99 to 3.34 (0.74–1.91) µg/g cr for those in county B. The predetermined benchmark response of 0.05 and the 90^th^ percentiles of urinary β2-microglobulin and N-acetyl-β-D-glucosaminidase levels of the subjects not exposed to cadmium (i.e., the control group) served as cut-off values.

**Conclusions:**

The obtained BMDs of urinary cadmium were similar to the reference point of 1 µg/g cr, as suggested by the European Food Safety Authority, indicating that cadmium exposure must be reduced to protect human health.

## Introduction

Cadmium (Cd) is a heavy metal that is toxic to human beings; however, it remains a widespread environmental pollutant primarily due to the agricultural and industrial use of Cd and its associated compounds [1], [2]. Cd-contaminated food and tobacco smoke are the major sources of Cd intake for the general population [3]. The kidney is particularly affected by Cd, as even low-level Cd exposure can result in renal tubular damage, which is characterized by increased urinary excretion of low-molecular-weight proteins or intracellular tubular enzymes [4]–[6]. To prevent these negative effects of Cd on human health, a reference level for Cd should be established based on sensitive indices of Cd exposure-related health effects. The Cd content in urine (U-Cd) is an index of internal Cd burden, and dose-response relationships between U-Cd and indicators of renal dysfunction, such as urinary β2-microglobulin (U-β_2_M) concentration and N-acetyl-β-D-glucosaminidase activity (U-NAG), have been found among people in both Cd-polluted and non-polluted areas [7]–[12]. Various methods have been used to estimate the U-Cd thresholds for Cd-induced renal tubular effects and have demonstrated large variations among people in different countries (0.3–10 µg/g cr) [3], [13].

The benchmark dose (BMD) is defined as the exposure level corresponding to a certain increase in the probability of an adverse response (i.e., benchmark response [BMR]; e.g., 1%–10%) compared with zero background exposure [14], [15]. For example, a BMD could represent a 95% statistical lower confidence limit on the dose corresponding to a 1% increase in an adverse response over that found with zero background exposure. The benchmark level of an adverse change in response (BMR) would thus be 1% in this example. The lower 95% confidence limit of BMD (BMDL) has been suggested to replace the no observed adverse effect level (NOAEL) [14]–[16]. The BMD/BMDL approach, which corresponds to a certain response or risk level, uses the whole dose-response curve [15]. Thus, BMD/BMDL is based on more information than the NOAEL. The BMD method has been widely used and has been applied to the health risk assessment of environmental contaminants (cadmium, lead, acrylamide, ethylene glycol monopropyl ether, etc.) [15]. However, this approach has not been appropriately applied to assess Cd exposure in the Chinese population, particularly among women.

Women are at high risk of suffering from Cd-related health effects [5], [17]–[19]. In the present study, we included non-smoking women aged 35–54 in two typical Cd-polluted areas in China and their corresponding controls (i.e., women from non-Cd-polluted areas). All participants in the present study engaged in farming as an occupation and did not have occupational Cd exposure. BMDs and BMDLs of U-Cd were tested for Cd-induced kidney effects to provide evidence for the prevention and control of environmental Cd exposure.

## Materials and Methods

This research was approved by the Ethics Committee of Tongji Medical College of Huazhong University of Science and Technology (IORG No: IORG0003571). The participants were informed about the purpose and methods of the study, and each participant provided written informed consent. This study was based on data collected through two epidemiological investigations, one of which was launched in county A (CA) in 2011, and the other of which was launched in county B (CB) in 2006.

### Study Area

CA and CB are both located in south-central China. In 1980, a smelter was established in CA. This smelter was the largest in this area during the study period, with an annual copper production of approximately 400,000 tons. Over the past 20 years, industrial wastewater was used for irrigation of local farmlands, resulting in serious Cd pollution of soils and contamination of rice crops. Three villages approximately 2–3 km on the leeward side of the smelter (Cd-polluted areas) and two comparable villages approximately 10 km in the opposite direction from the smelter (controls) were selected. A non-ferrous smelter was established in CB in 1960. Three villages in CB were selected, including two villages located approximately 2–4 km on the leeward side of the smelter (Cd-polluted areas) and one comparable village approximately 30 km in the opposite direction from the smelter (control).

### Study Population

Female non-smokers aged 35–54 years and without any occupational Cd exposure were recruited in the two studied regions. All the subjects were randomly selected using stratified cluster sampling. First, villages were randomly selected in every county that was included in study area; thereafter, using a table of random permutations and digits, subjects were selected from every village according to the age group. All the subjects lived in the studied areas for over five years and mainly subsisted on locally grown crops. They mainly engaged in farming as an occupation. Those exposed to occupational Cd (or other toxic metals, such as lead and mercury) were excluded. Smokers were also excluded. A questionnaire designed by our team specifically for this study was used to collect data on the demographics of all participants, and the entire questionnaire was administered by interviewers.

### Sample Collection and Analysis

Morning urine samples (first sample after the first morning void) of approximately 50–100 mL were collected from all subjects. The samples were collected in 250 mL polyethylene bottles that were soaked in 3 mol/L nitric acid for 16 h and rinsed in deionized water prior to ruse. Each sample was then divided equally and stored in three tubes, one of which had crystalline sodium carbonate on the tube wall for pH adjustment to stabilize U-β_2_M. The second tube was used for Cd analysis and the third tube was used for the determination of U-NAG activity and creatinine (Cr) concentration. All urine samples were stored and frozen at −80°C until further analysis. U-β_2_M was detected through radioimmunoassay, whereas U-Cd concentration was determined by graphite-furnace atomic absorption spectrometry (SpectrAA-240FS; Varian, Palo Alto, CA, USA) [20]. The detection limit of Cd in urine was 0.05 µg/L, and none of the subjects had values below this limit. The relative standard deviation of the precision test was 4.5–7.9% (n = 5), and the recovery of standard addition was 94.8–98.3%. U-β_2_M kits were purchased from the Chinese Academy of Science. U-NAG was measured as previously described by Tucker et al. [20], [21]. Substrate and standard were purchased from Sigma. The amount of urinary Cr was determined by using a modified Jaffe reaction method [22]. Concentrations of urinary substances were expressed in corrected Cr units (/g cr). The levels of U-Cd and U-β_2_M were expressed in units of µg/g cr, and U-NAG was expressed in units of U/g cr. The accuracy of each analysis was evaluated using duplicate measurements. All samples were tested in duplicate.

### Statistical Analysis

The levels of the three urinary Cd substances were confirmed to fit a log-normal distribution. Thus, geometric mean (GM) and geometric standard deviation (GSD) were adopted to represent the distributions. Age was presented as the arithmetic mean and standard deviation. Differences in age and U-Cd, U-β_2_M, and U-NAG values between the two groups were analyzed using the Student's *t*-test. The chi-square test was used to analyze the significance of the linear trend in Cd-induced kidney damage. Linear regression was used to analyze the relationship among the three urinary substances. In the multiple regression models, U-β_2_M and U-NAG, which are two sensitive indicators of renal tubular dysfunction, were regarded as dependent variables, whereas age and U-Cd were considered as explanatory variables. In the logistic regression model, U-β_2_M and U-NAG were employed as criterion variables (categorical variables), whereas age and urinary Cd excretion were considered as explanatory variables (continuous variables). The analyses were conducted using SPSS version 18 (SPSS Inc., Chicago, IL, USA).

The corresponding 84^th^ and 90^th^ percentiles of Cd effect indicators of subjects living in the control areas were regarded as the cut-off values, as shown in Table 1. The values of BMD and BMDL were calculated using the Benchmark Dose Software (version 2.0) provided by the U.S. Environmental Protection Agency. An extra type of BMR of 0.05 or 0.10 was adopted in the BMD application. The BMD and BMDL values were calculated using a quantal-linear model as follows: P[response]  =  background + [1 – background] × [1 – EXP (– slope × dose)].

**Table 1:** Cut-off values of U-β_2_M and U-NAG for subjects in county A (CA) and county B (CB).

Variable		Region	Cut-off value		
			N	84^th^ percentile	90^th^ percentile
U-β_2_M (µg/g cr)		CA	130	1065.95	1541.33
		CB	116	1045.96	1268.95
U-NAG (U/g cr)		CA	130	5.67	6.42
		CB	116	40.90	53.38

U-β2M, urinary level of β2-microglobulin; U-NAG, urinary level of N-acetyl-β-D-glucosaminidase

## Results

A total of 490 subjects were enrolled in the study, including 130 in the CA control group and 139 in the CA Cd-polluted group, as well as 116 in the CB control group and 105 in the CB Cd-polluted group. The mean ages and concentrations of U-Cd, U-NAG, and U-β_2_M of the subjects grouped by residence are shown in Table 2. In CA, the mean age of the control group was 44.35 years, which was not significantly different from that of the Cd-exposed group (44.06 years; *t* = 0.389; *P* = 0.698). The GM (GSD) of U-Cd was 7.00 (2.22) µg/g cr in the Cd-exposed group, which was significantly higher than that in the control group (2.69 [2.31] µg/g cr; *t* = 9.608, *P*<0.01). The GM (GSD) of U-NAG in the Cd-exposed group was also higher than that of the control group (4.04 vs. 2.65 U/g cr; *t* = 4.251; *P*<0.05), whereas the difference in the U-β_2_M level between the two groups did not reach significance (*t* = 0.050; *P* = 0.618). In CB, the mean ages of the control and Cd-exposed groups were 44.38 and 45.39 years, respectively (*t* = −1.245, *P* = 0.214). The GMs (GSDs) of U-Cd and U-β_2_M were 1.25 (2.16) µg/g cr and 584.72 (1.77) µg/g cr in the control group, respectively, which were notably lower than the counterpart values of 6.83 (2.20) µg/g cr and 954.09 (1.88) µg/g cr in the Cd-exposed group (U-Cd: *t* = −16.215; *P*<0.01; U-β2M: *t* = −6.063; *P*<0.01). No significant difference in U-NAG activities was observed between the two groups (*t* = 1.398, *P* = 0.164). Multiple linear regression analysis and logistic regression analysis showed that U-β_2_M and U-NAG were positively associated with U-Cd among CA and CB subjects, and age was not associated with either U-β_2_M or U-NAG (as shown in Tables 3 and 4).

**Table 2:** Means (SD/GSD) of age, U-Cd, U-β2M, and U-NAG of 269 participants in county A (CA) and 225 participants in county B (CB) △.

Characteristic or indicator	CA		CB	
	Control group (n = 130)	Exposed group (n = 139)	Control group (n = 116)	Exposed group (n = 105)
Age (year)	44.35 (6.35)	44.06 (5.85)	44.38 (6.00)	45.39 (5.96)
U-Cd (µg/g cr)	2.69 (2.31)	7.00* (2.22)	1.25 (2.16)	6.83* (2.20)
U-β_2_M (µg/g cr)	454.26 (2.57)	479.39 (2.27)	584.72 (1.77)	954.09* (1.88)
U-NAG (U/g cr)	2.65 (2.49)	4.04* (2.05)	13.43 (2.73)	16.49 (3.23)

△ Values of U-Cd, U-β_2_M, and U-NAG are expressed as GM and GSD, and values of age were calculated by arithmetic mean and SD.

*Compared with the control group, *P*<0.01.

U-β2M, urinary level of β2-microglobulin; U-NAG, urinary level of N-acetyl-β-D-glucosaminidase; U-Cd, urinary level of cadmium; GM, geometric mean; GSD, geometric standard deviation; SD, standard deviation.

**Table 3:** Results of the multiple linear regression analysis on individual factors related to Cd-induced renal tubular damage.

Region	Effect markers	Explanatory variables	B^2^	95% CI^3^	Beta^4^	*P*-value
CA	U-β_2_M (µg/g cr)^1^	U-Cd (µg/g cr) 1	0.159	(0.047, 0.270)	0.170	<0.05
		Age (years)	0.006	(−0.001, 0.014)	0.057	0.110
	U-NAG (U/g cr)^1^	U-Cd (µg/g cr) 1	0.257	(0.153, 0.361)	0.290	<0.001
		Age (years)	−0.001	(−0.008, 0.006)	-0.013	0.833
CB	U-β_2_M (µg/g cr)^1^	U-Cd (µg/g cr) 1	0.292	(0.227, 0.356)	0.519	<0.001
		Age (years)	0.001	(−0.004, 0.007)	0.027	0.646
	U-NAG (U/g cr)^1^	U-Cd (µg/g cr) 1	0.261	(0.140, 0.381)	0.276	<0.001
		Age (years)	0.010	(0.000, 0.020)	0.131	0.063

1 U-β_2_M, U-NAG and U-Cd were natural log-transformed.

2 B: Un-standardized partial regression coefficients.

3 CI: Confidence interval.

4 Beta: Standardized partial regression coefficients.

CA, county A; CB, county B; U-β2M, urinary level of β2-microglobulin; U-NAG, urinary level of N-acetyl-β-D-glucosaminidase; U-Cd, urinary level of cadmium.

**Table 4:** Results of the logistic regression analysis on individual factors related to Cd-induced renal tubular damage.

Region	Effect markers	Explanatory variables^5^	84^th^ cutoff^1^	90^th^ cutoff^2^
			OR^3^ (95% CI^4^)	OR^3^ (95% CI^4^)
CA	U-β_2_M (µg/g cr)	U-Cd (µg/g cr)	1. (1.011, 1.100)	1.056* (1.008, 1.107)
		Age (years)	1.040 (0.986, 1.096)	1.014 (0.952, 1.080)
	U-NAG (U/g cr)	U-Cd (µg/g cr)	1.095** (1.050, 1.143)	1.097** (1.050, 1.145)
		Age (years)	0.993 (0.946, 1.041)	0.992 (0.940, 1.046)
CB	U-β_2_M (µg/g cr)	U-Cd (µg/g cr)	1.197** (1.124, 1.274)	1.156** (1.095, 1.220)
		Age (years)	1.003 (0.949, 1.060)	0.991 (0.932, 1.054)
	U-NAG (U/g cr)	U-Cd (µg/g cr)	1.074** (1.024, 1.126)	1.075** (1.019, 1.135)
		Age (years)	1.008 (0.949, 1.070)	1.063 (0.985, 1.148)

1, 2 Cut-off values of U-β_2_M or U-NAG were defined as the 84^th^ or 90^th^ percentile values, which were calculated in subjects from Cd-non-polluted areas.

3 OR: Odds ratio.

4 CI: Confidence interval.

5 Explanatory variables were used as continuous variables.

**P<0.01, *P<0.05.

CA, county A; CB, county B; U-β2M, urinary level of β2-microglobulin; U-NAG, urinary level of N-acetyl-β-D-glucosaminidase; U-Cd, urinary level of cadmium.

To further elucidate the relationship between the indicators of renal function (U-β_2_M and U-NAG) and Cd exposure, U-β_2_M and U-NAG concentrations were compared among U-Cd intervals grouped based on the three corresponding quartiles. As shown in Tables 5 and 6, an obvious increase in U-β_2_M and U-NAG occurred as U-Cd increased. As shown in Table 5, the 84^th^ percentiles of U-β_2_M and UNAG of the control group were established as the thresholds of hyper-U-β_2_M and hyper-U-NAG, respectively. As shown in Table 6, the 90^th^ percentiles of U-β_2_M and UNAG of the control group were established as the thresholds of hyper-U-β_2_M and hyper-U-NAG, respectively.

**Table 5:** Prevalence of hyper-U-β_2_M and hyper-U-NAG corresponding to U-Cd intervals among subjects in county A (CA) and county B (CB) at different cadmium exposure levels^1^.

U-Cd intervals (µg/g cr) 2	CA						CB			
		Hyper-U-NAG		Hyper-U-β_2_M			Hyper-U-NAG		Hyper-U-β_2_M	
	Median (U-Cd) (µg/g cr)	+/−	%	+/−	%	Median (U-Cd) (µg/g cr)	+/−	%	+/−	%
<Q_1_	1.44	11/56	16.41	5/62	7.46	0.72	3/52	5.45	6/49	10.91
Q_1_–Q_2_	3.43	8/60	11.76	9/59	13.24	1.72	10/45	18.18	10/45	18.18
Q_2_–Q_3_	5.97	20/47	29.85	16/51	23.88	3.80	8/48	14.28	13/43	23.21
>Q_3_	13.80	30/37	44.78	18/49	26.86	12.36	17/38	30.90	35/20	65.45
Total	4.47	69/200	25.65	48/221	17.84	2.86	38/183	17.19	64/157	28.96
Linear trend test		χ^2^ = 17.622, *P*<0.001		χ^2^ = 10.807, *P* = 0.001			χ^2^ = 10.073, *P* = 0.002		χ^2^ = 35.370, *P*<0.001	

1 The 84^th^ percentiles of U-β_2_M and U-NAG values of the control group were assumed as the thresholds of hyper-U-β2M and hyper-U-NAG, respectively.

2 U-Cd intervals were divided according to the three corresponding quartiles (i.e., Q_1_ = 2.38 µg/g cr_,_ Q_2_ = 4.47 µg/g cr, and Q_3_ = 8.59 µg/g cr in CA; and Q_1_ = 1.16 µg/g cr_,_ Q_2_ = 2.86 µg/g cr, and Q_3_ = 6.88 µg/g cr in CB).

U-β2M, urinary level of β2-microglobulin; U-NAG, urinary level of N-acetyl-β-D-glucosaminidase; U-Cd, urinary level of cadmium.

**Table 6:** Prevalence of hyper-U-β_2_M and hyper-U-NAG corresponding to U-Cd intervals among subjects from county A (CA) and county B (CB) at different cadmium exposure levels^1^.

U-Cd intervals (µg/g cr) 2	CA					CB				
		Hyper-U-NAG		Hyper-U-β_2_M			Hyper-U-NAG		Hyper-U-β_2_M	
	Median of U-Cd (µg/g cr)	+/−	%	+/−	%	Median of U-Cd (µg/g cr)	+/−	%	+/−	%
<Q_1_	1.44	8/59	11.94	3/64	4.08	0.72	2/53	3.64	2/53	3.64
Q_1_–Q_2_	3.43	5/63	7.35	6/62	6.67	1.72	6/49	10.91	8/47	14.55
Q_2_–Q_3_	5.97	11/56	16.42	10/57	17.39	3.80	3/53	5.36	8/48	14.28
>Q_3_	13.80	24/43	35.82	12/55	15.09	12.36	12/43	21.82	27/28	49.10
Total	4.47	48/221	17.84	31/238	11.52	2.86	23/198	10.41	45/176	20.36
Linear trend test		χ^2^ = 14.876, *P*<0.001		χ^2^ = 7.055, *P* = 0.008			χ^2^ = 7.015, *P* = 0.008		χ^2^ = 31.194, *P*<0.001	

1 The 90^th^ percentiles of U-β_2_M and U-NAG of the control group were assumed as the thresholds of hyper-U-β2M and hyper-U-NAG, respectively.

2 U-Cd intervals were divided according to the three corresponding quartiles (i.e., Q_1_ = 2.38 µg/g cr_,_ Q_2_ = 4.47 µg/g cr, and Q_3_ = 8.59 µg/g cr in CA; and Q_1_ = 1.16 µg/g cr_,_ Q_2_ = 2.86 µg/g cr, and Q_3_ = 6.88 µg/g cr in CB).

U-β2M, urinary level of β2-microglobulin; U-NAG, urinary level of N-acetyl-β-D-glucosaminidase; U-Cd, urinary level of cadmium.

The BMD and BMDL results obtained using the quantal-linear model with the predetermined cut-off values and BMRs of 0.05 or 0.10 are shown in Table 7. Corresponding to a predetermined BMR of 0.10, with the 84^th^ and 90^th^ percentiles of the control group defined as the cut-off values, the U-Cd BMDLs based on U-NAG for the CA subjects were 2.12 and 2.90 µg/g cr, respectively, and those for the CB subjects were 2.65 and 3.92 µg/g cr, respectively. Corresponding to a predetermined BMR of 0.05, with the 84^th^ and 90^th^ percentiles of the control group defined as the cut-off values, the U-Cd BMDLs based on U-NAG for subjects in CA were 1.03 and 1.41 µg/g cr, respectively, and those for subjects in CB were 1.29 and 1.91 µg/g cr, respectively. Corresponding to a predetermined BMR of 0.10, with the 84^th^ and 90^th^ percentiles of the control group defined as the cut-off values, the U-Cd BMDLs based on U-β_2_M for CA subjects were 3.06 and 4.48 µg/g cr, respectively, and those for CB subjects were 1.09 and 1.51 µg/g cr, respectively. Corresponding to a predetermined BMR of 0.05, with the 84^th^ and 90^th^ percentiles of the control group defined as the cut-off values, the U-Cd BMDLs based on U-β_2_M for subjects in CA were 1.49 and 2.18 µg/g cr, respectively, and those for subjects in CB were 0.53 and 0.74 µg/g cr, respectively.

**Table 7:** Benchmark dose (BMD) estimates of U-Cd levels based on U-β_2_M and U-NAG using a quantal-linear model^1^.

Region	Effect markers	Cut-off 2	Background	Slope	AIC	BMD_10_/BMDL_10_ 3	BMD_5_/BMDL_5_ 3	*P*-value
CA	U-NAG (U/g cr)	84^th^	0.092	0.035	291.53	3.05/2.12	1.48/1.03	0.08
		90^th^	0.051	0.025	239.51	4.27/2.90	2.08/1.41	0.19
	U-β_2_M (µg/g cr)	84^th^	0.066	0.022	246.67	4.89/3.06	2.38/1.49	0.30
		90^th^	0.040	0.014	189.80	7.80/4.48	3.80/2.18	0.52
CB	U-NAG (U/g cr)	84^th^	0.080	0.024	196.39	4.39/2.65	2.14/1.29	0.18
		90^th^	0.041	0.015	143.05	6.86/3.92	3.34/1.91	0.22
	U-β_2_M (µg/g cr)	84^th^	0.057	0.072	227.15	1.46/1.09	0.71/0.53	0.67
		90^th^	0.014	0.052	190.92	2.03/1.51	0.99/0.74	0.31

1 Quantal-linear model: *P*[response]  =  background + [1 – background] × [1 – EXP (– slope × dose)].

2 The cut-off value is either the 84^th^ or 90^th^ percentile of U-β_2_M or U-NAG levels in Cd non-exposed subjects (i.e., the control group).

3 BMD_10_ and BMDL_10_ values correspond to a predetermined BMR of 0.10; BMD_05_ and BMDL_05_ values correspond to a predetermined BMR of 0.05; BMDL is the lower 95% confidence limit of BMD.

U-β2M, urinary level of β2-microglobulin; U-NAG, urinary level of N-acetyl-β-D-glucosaminidase; U-Cd, urinary level of cadmium; CA, county A; CB, county B; BMD, benchmark dose; BMDL, 95% lower confidence bounds of benchmark dose.

## Discussion

The negative effects of Cd on human health are typically manifested in the kidney. Cd exposure can cause both tubule and cellular damage in the kidney, which is characterized by the increased excretion of low-molecular-weight proteins, such as U-β_2_M, and intracellular tubular enzymes, such as U-NAG [23]–[25]. Thus, the levels of these two substances in urine were selected as indicators of renal function in this study.

Previous studies revealed that U-Cd values were associated with factors such as gender, age, smoking habits, and occupational exposure. Women were reportedly more susceptible to Cd exposure than men, with higher Cd contents observed in the urine and kidney cortex of women [19], [26], [27]. In addition, this trend may be attributed to increased dietary Cd absorption in women with low iron stores due to menstruation [28]–[30]. Smokers were found to have U-Cd concentrations approximately two to five times higher than that of nonsmokers [5], [31]–[33]. Suwazono et al. suggested that individual age should be considered when estimating the threshold value of U-Cd [34]. Thus, in this study, non-smoking women aged 35–54 years and without occupational Cd exposure were included. Women of this age were at a high risk of Cd exposure. According to our findings, age was not associated with U-NAG and U-β_2_M. We speculated that the variable of age did not show any effect because of the narrow age range of participants included in this study. After controlling for age, the levels of U-β_2_M and U-NAG were found to be significantly associated with U-Cd concentrations. Similar dose-response relationships between U-Cd and indicators of renal function were reported in many other studies conducted on Cd-polluted or non-polluted areas [7]–[9], [11].

In BMD estimations, we regarded the subjects living in Cd-non-polluted areas as controls. Both controls in CA and CB were exposed to Cd at low levels, similar to a previous study [7], and thus, these women were not strict non-exposed controls. Cd exposure in Asia (e.g., China, Japan) is more serious than that in Europe and the United States. The rapid development of polluting industries has resulted in contamination of soils with Cd, thereby leading to contamination of field crops with Cd. Therefore, people may have a diet with some Cd intake [35]. Unlike in animal experiments, a perfect control with no Cd exposure seems impossible in population studies. Contrary to NOAEL, the BMD method does not need to calculate the abnormality rate for the control subjects, which is an important advantage of the BMD approach [13], [34]. The probability of adverse response at zero exposure was estimated based on the cut-off value of the control group. The exposure level was then calculated in such a way that a certain change in response compared with the background was expected. In this sense, the BMD/BMDL calculations were more accurate and provided more information. The use of different models and points to study the BMD for Cd can also provide more evidence for the control and prevention of human Cd exposure. In the BMD procedure, we used the quantal-linear model, which has been used in previous risk assessments of Cd exposure [36]. In addition, the most important reason for using BMD is that the model provides a good fit to the present data (results using other models not shown). Therefore, the quantal-linear model was selected to estimate the critical concentration of U-Cd in this study.

U-β2M and U-NAG were measured as sensitive indicators of renal tubular damage in our study. Slightly increased urinary excretion of U-β2M between 300 and 1000 µg/g cr might be reversible after removal of Cd exposure [37]–[39]. However, this assumption remains under debate. Liang et al. investigated the change in renal function after reducing Cd exposure. In this study, 412 individuals were examined in 1998 and eight years later. The study revealed that U-β2M increased from 120–160 µg/g cr in non-Cd-polluted areas, from 160–280 µg/g cr in moderate-Cd-polluted areas and from 280–420 µg/g cr in high-Cd-polluted areas [35]. Nevertheless, a consensus exists that severely increased urinary excretion of U-β2M over 1000 µg/g cr is irreversible. Kido et al. conducted a five-year follow-up study and found that almost all individuals who had U-β2M levels over 1000 µg/g cr in the first examination exhibited renal deterioration [40]. Other researchers also confirmed that the critical value of reversible U-NAG has yet to be fully elucidated [40]–[44]. In this study, we adopted the 84th and 90th percentiles of renal function indicators (i.e., U-β2M and U-NAG) of the non-exposed subjects as cut-off values. The 84th percentiles of U-β2M were 1065.95 µg/g cr and 1045.96 µg/g cr for the control subjects in CA and CB, respectively. The values were nearly equal to the robust irreversible value of 1000 µg/g cr. The cut-off values estimated from the Cd-non-exposed subjects were slightly higher than those reported in Europe and the United States, the reasons for which have been discussed above.

According to the studies of Hayano et al. and Ishizaki et al., the U-Cd thresholds were 3.8–4.1 µg/g cr and 2.3–4.6 µg/g cr based on the U-β2M values of subjects living in the Cd-polluted Kakehashi River basin [7]. Shimizu et al. estimated the U-Cd threshold to be 1.5–3.6 µg/g cr based on the U-β2M excretions of 1865 women in Cd-polluted regions of the Kakehashi River basin in Japan [36]. Kobayashi et al. reported a U-Cd threshold of 1.6 µg/g cr based on the U-β2M values of 723 women in a Cd-non-polluted area in Japan [45]. Hong et al. conducted a study involving a general population in China and determined that the BMD/BMDL of U-Cd was 1.05/0.88 µg/g cr with a predetermined BMR of 10% [46]. Suwazono et al. presented a low U-Cd BMD (BMDL) of 0.6–1.1 (0.5–0.8) µg/g cr based on the U-NAG values of 820 Swedish women aged 53–64 years [13]. In the present study, the BMDL values of U-Cd based on U-β_2_M were 1.84 µg/g cr in CA and 0.57 µg/g cr in CB, with the 84^th^ percentiles designated as cut-off values, whereas the BMDL values of U-Cd based on U-NAG were 1.19 µg/g cr in CA and 1.37 µg/g cr in CB, with the 84th percentiles designated as cut-off values. In our previous report, a different reference group (i.e. those with U-Cd of <2.00 µg/g cr) was chosen, and the BMD approach was adopted by combining both sets of data from CA and CB subjects, to present either LogProbit or LogLogistic Models according to the AIC values. The U-Cd levels were divided into intervals of <2.00 µg/g cr, 2.01–4.00 µg/g cr, 4.01–10.00 µg/g cr, and >10.00 µg/g cr [47]. The findings of both reports are similar to those of Suwazono et al. and Jin et al. [13], [46]. The results of our study and other studies suggest an increased health risk with U-Cd levels exceeding 1 µg/g cr.

An interesting finding of this study was that the cut-off points for U-NAG (i.e., the 84th percentile of 5.62 U/g cr in the CA population and 40.96 U/g cr in the CB population, as well as the 90th percentile of 6.42 U/g cr in the CA population and 53.38 U/g cr in the CB population) were significantly different. Although the methods used for U-NAG detection, including the testing technology and equipment, were the same, the reagents used in the analysis had different batch numbers. We found that during the CB investigation, all urine samples were immediately divided into several parts for various measurements after their collections. All samples were then transported in cold chain to our institution for laboratory tests. All samples were stored at −80°C until analysis. During the CA investigation, all urine samples were collected and soon transported in cold chain to our institution. The samples were then divided to several parts and stored at −80°C until analysis. The CB area is nearer (with a distance of approximately 110 km) to our institution than the CA area (approximately 520 km). We believe that the most plausible reason for the difference in results for U-NAG cut-off values was that the cold chain was not completely ensured during the long journey from CA to our institution, and hence, the activities of U-NAG in CA urine samples may have been impaired to some extent. Thus, we reconsidered the comparability of the two sets of data from CA and CB, respectively. We believe that direct comparison was unsuitable. For the stability of U-Cd, we selected two representative controls for CA and CB data. Moreover, in this research, uncertainty existed in the test results for U-NAG, which may not be a stable effect marker. This condition is also a reminder to other researchers that more attention should be given to this problem. Nevertheless, our study can provide some scientific evidence to aid in the decision-making of governmental departments responsible for supervising environmental Cd pollution and public health. The data could be used for scientific exploration but are insufficient for the clinical diagnosis of Cd-induced renal damage.

In conclusion, the threshold level of U-Cd in our study was estimated in two typical Cd-polluted areas, with a sample population of women aged 35–54 years. This population has been seldom considered in Chinese studies. We believe that the BMDs/BMDLs of U-Cd, which were based on the participants most sensitive to Cd, may be used to establish a reasonable safety level for Cd to protect the entire population. The BMDL value was inferred to be almost the same as those of other ages in Japan and Sweden. Our study findings confirm the importance of decreasing Cd exposure levels to protect human health.
